# Functional outcomes in early (T1/T2) supraglottic cancer: a systematic review

**DOI:** 10.1186/s40463-018-0321-8

**Published:** 2018-12-18

**Authors:** Benjamin van der Woerd, Krupal B. Patel, Anthony C. Nichols, Kevin Fung, John Yoo, S. Danielle MacNeil

**Affiliations:** 1 Department of Otolaryngology, Head & Neck Surgery, Schulich Medicine & Dentistry, Western University, London Health Sciences Centre, Victoria Hospital, London, Ontario Canada; 2 Department of Otolaryngology, Head and Neck Surgery, The Ohio State University, Columbus, OH USA

**Keywords:** Early stage, Supraglottic squamous cell carcinoma, Supraglottic SCC, Outcomes, Systematic review, Functional outcomes

## Abstract

**Objectives:**

Organ preserving surgery (OPS) and radiotherapy (RT) are both accepted treatment options for early stage supraglottic cancer (SGC). Radiation has supplanted surgery in most cases, because of the perception that surgery results in poorer functional outcomes. However, evidence suggests that OPS with a neck dissection may be associated with improved survival. Our objective was to conduct a systematic review of the literature to compare functional outcomes of OPS and RT for early SGC.

**Methods:**

We searched Medline, EMBASE and Cochrane Central Register of Controlled Trials to identify studies. Studies were included if they reported functional outcomes on 10 or more patients with early stage SGC treated with radiation or OPS, including open partial laryngectomy, transoral laser microsurgery (TLM) or transoral robotic surgery (TORS). Two reviewers independently screened articles for relevance using pre-determined criteria.

**Results:**

From 7720 references, we included 10 articles (*n* = 640 patients). 50% (*n* = 320) of patients were treated with surgery. Three head-to-head RT versus OPS papers were included, however different outcome measures were used for each group. Intractable aspiration management (including total laryngectomy or permanent tracheostomy) following OPS was reported in five papers representing 186 patients; the definitive intractable aspiration management rate was 2.6% (95% CI 1.0–6.8%). Four papers reported permanent G-tube rate for the surgical group (*n* = 198), calculating a rate of 5.3% (95% CI 2.6–10.5%), this was not reported for the RT group in any papers. One study reported quality of life. Two studies reported objective voice measures.

**Conclusions:**

This systematic review revealed a paucity of objective measures and significant data heterogeneity, rendering the comparison of functional outcomes following OPS versus RT for early SGC limited. Future research should include objective measures of functional outcomes including laryngectomy rate, g-tube rate, tracheostomy dependence, quality of life, and voice quality measures.

## Introduction

Early stage supraglottic squamous cell carcinoma (SSCC) includes T1 tumours, isolated to one subsite of the supraglottis with normal vocal cord function, or T2 tumours, involving more than one subsite of the supraglottis, glottis, or surrounding tissue, without evidence of regional disease spread [1]. A recent study of nearly 160,000 laryngeal cancer SCC patients in the United States, found the incidence of SSCC to account for one third of laryngeal cancers [2]. Laryngeal cancers are the most common malignant lesions of the head and neck, with an estimated 13,150 new laryngeal cancers per year [3].

The recommendations from the National Comprehensive Cancer Network (NCCN) guidelines for treatment of early stage SSCC include both organ preservation strategies – radiation (RT) or organ preservation surgery (OPS) with or without a neck dissection [3, 4]. Despite small tumour sizes, 5-year survival for early stage SSCC is 64%, and oncologic outcomes have not improved over the past 30 years [2, 5–8]. This is hypothesized to be a result of the robust lymphatic supply to the supraglottis leading to higher rates of occult metastases to regional and distant sites. When comparing the relative 5-year survival from 1985 to 1987 to 1994–1996, there was a decline from 52.2 to 47.3%. Reviewing the data from the National Cancer Database, the largest decline was identified in patients with T1 N0-T2 N0 disease. A recent meta-analysis by Patel et al. (2018) examining survival in early stage supraglottic SCC suggested that primary surgery may result in decreased disease specific survival (OR 0.43, 95% CI 0.31–0.60) and overall mortality (OR 0.40, 0.29–0.55) when compared with primary radiotherapy [9].

There are a limited number of studies, with no previous meta-analysis, that have compared the functional outcomes between primary surgery and primary radiotherapy for early stage SSCC. Much of the available data focuses on survival outcomes for the two treatment modalities [7–17]. There are no prospective clinical trials, and the majority of the studies reported are small and retrospective in design. Our objectives were to systematically review the literature to find all the relevant studies about the functional outcomes for surgery and radiation for early stage SSSC, synthesize the results and perform meta-analyses where possible.

## Methods

A systematic review protocol was developed a priori to ensure the objectives and aims were outlined from the outset.

Computerized bibliographic databases: Medline, EMBASE and Cochrane Central Register of Controlled Trials were searched to identify studies. English language records were included from January 1990 to October 2018. The search strategy was designed by three authors (B.V.W., K.B.P. and S.D.M.) and an experienced librarian.

Randomized controlled trials, head-to-head comparative studies, observational studies, and case series that included 10 or more patients were reviewed. Single arm studies that reported functional results of organ preservation surgeries or radiotherapy as single modality therapy were included in the review. Studies on organ preserving surgeries, including open partial laryngectomy, transoral laser microsurgery (TLM) or transoral robotic surgery (TORS), with and without neck dissection were included.

The study population was limited to patients aged 18 and older, diagnosed with early stage supraglottic SCC (Tis, T1 N0, T2 N0). We excluded studies where functional results for patients with advanced T stage or node positive disease were grouped into the results and could not be reliably differentiated. We also excluded studies where functional outcomes for patients with early supraglottic cancers were combined with early glottic cancers.

Included studies were assessed for the following functional outcomes: aspiration; gastrostomy tube dependence; objective voice outcomes; and quality of life measures.

Titles, abstracts, and full texts of the studies were reviewed independently by two authors (B.V.W. and K.B.P.). Disagreements were resolved by consensus. Inter-observer agreement was analyzed with Cohen’s kappa. Titles were screened for the keywords: “squamous cell carcinoma” and “supraglottic”, or “supraglottis”, or “glottic”, or “glottis”, or “larynx”, or “laryngeal”. All study abstracts that met the eligibility criteria were then screened individually. The full text of studies that met inclusion criteria were then reviewed. The Newcastle-Ottawa Quality Assessment Scale for Cohort Studies was used to determine the quality of the studies (Table 2) [18]. Relevant data was extracted using a standardized data extraction form. Not all studies contained data for each of the outcome measures.

Review Manager 5.3 and Comprehensive Meta-Analysis applications were used for statistical analysis. Dichotomous outcomes were compared using odds ratios (OR) or weighted incidence rates and 95% confidence intervals (CI). Heterogeneity across the studies was evaluated by the chi-square statistic and significance was set at *p* < 0.05. The I^2^ test was used to measure the extent of inconsistency across the results.

A random effects model was used to allow for differences in the treatment effects from study to study. The Z statistic was used to test for overall pooled effect and significance was set at p < 0.05.

## Results

The search strategy produced 7720 records. After duplicate records were removed from the search, 5218 unique records remained. After reviewing the titles, 1187 abstracts were deemed appropriate for abstract review. Following abstract review, 115 studies were appropriate for full text review. Ten studies met the final inclusion criteria after reviewing the full text. CONCLUSIONS:

There is an overall paucity of literature available regarding functional outcomes in the treatment of early stage SSCC patients, the majority of which is moderate in overall quality and retrospective in nature. The majority of the data does not reflect the treatment algorithms of today, with the introduction of focused radiation therapies and endoscopic surgical approaches. Changes to treatment algorithms have not been reflected in head-to-head studies examining the functional outcomes. The outcome measures reported have significant heterogeneity in the current literature, which limits the ability to draw definitive conclusions.

Moving forward, we propose that aspiration, intractable aspiration intervention, permanent gastrostomy tube requirements, and objective quality of life scales as objective measures that should be included in future research on this topic. Future studies and research should include well designed prospective trials with rigorous reporting of outcome measures.

**Fig. 1:**
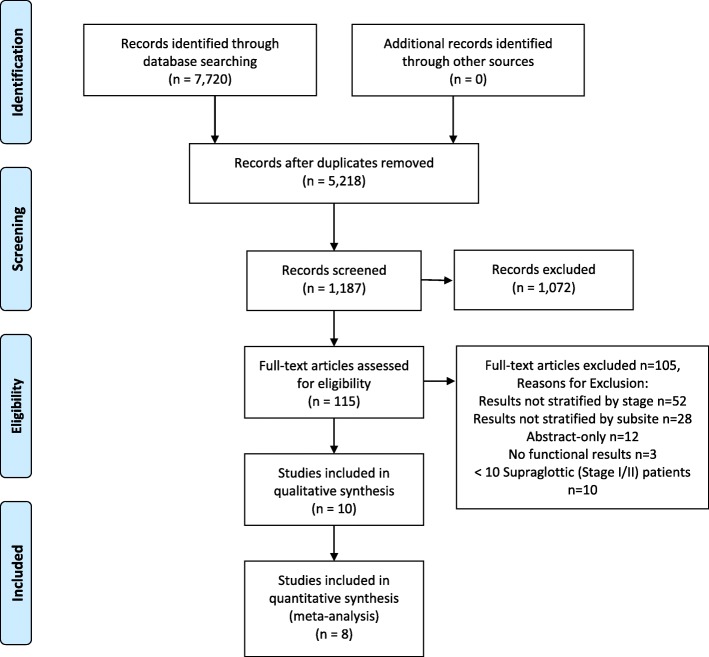
PRISMA Flowchart

Figure 1 Illustrates the PRISMA (Preferred Reporting Items for Systematic Reviews and Meta-Analyses) flow chart to identify the appropriate studies. Kappa statistic for the agreement at the abstract screening stage was 0.61 (95% CI 0.37–0.85) indicating moderate agreement.

### Study characteristics and methodologic quality

There were no randomized controlled trials comparing the functional outcomes of primary surgery versus radiation. Of the 10 studies included in the analysis, 6 were retrospective cohort studies, 4 were case series. There were no prospective studies included. There were 320 patients treated with OPS, including open partial laryngectomy, transoral laser microsurgery (TLM) or transoral robotic surgery (TORS). For the RT arm, there were 320 patients. Altogether, 640 patients were analyzed from 10 studies. There were 6 patients with early SSCC where the treatment modality was not specified and could not be included in the analyses. Characteristics of the included studies are summarized in Table 1. Overall, the quality of the included studies was moderate, this was for a variety of reasons, including no reference group (surgery or radiation arm only), short or unreported length of follow up, and unaccounted for patients. Table 2 summarizes the quality of the individual studies.

**Table 1:** Study Characteristics

Study ID	Location	Dates of Accrual	Age Mean (range)		OPS	RT	Stage
Ambrosch 2018	Italy	2002–2012	Median^c^ 62 (33–88)		27	0	T1 N0 (*n* = 8) T2 N0 (*n* = 19)
Bhattacharyya 2014	India	2006–2009	Median^c^ 59 (31–80)		0	10	T1 N0 (*n* = 1) T2 N0 (*n* = 9)
Carta 2018	Italy	2010–2017	Mean 61.8 (43–84)		27	0	T1 N0 (*n* = 11) T2 N0 (*n* = 16)
Chiesa Estomba 2015	Spain	2009–2012	Mean 64 (45–88)		72	0	T1 N0 (*n* = 44) T2 N0 (*n* = 28)
Chun 2010	South Korea	1991–2005	Mean 65.5		25	10	T1 N0 (*n* = 14) T2 N0 (*n* = 21)
Karatzanis 2010	Germany	1970–2004	Mean 60 (36–83)		78	0	T1 N0 (*n* = 29) T2 N0 (*n* = 49)
Mendenhall 1996	USA	1964–1992	NR		0	99	T1^b^ (*n* = 18) T2 (*n* = 81)
Oridate 2009	Japan	2006–2007	Median^c^ 76 (45–90)		NR^a^	11	T2 N0 (n = 11)
Orus 2000	Spain	1984–1996	Mean 60.5		25	90	T1 N0 (*n* = 38) T2 N0 (*n* = 77)
Spriano 1997	Italy	1983–1992	NR		66	100	T1 N0 (*n* = 112) T2 N0 (*n* = 54)

^a^6 patients not accounted for in the results, ^b^nodal staging not reported, ^c^Median age presented

**Table 2:** Newcastle-Ottawa quality assessment scale for cohort studies

Study	Selection	Comparability	Outcomes	Total Score
Ambrosch 2018^e^	3	2	3	8
Bhattacharyya 2014^b^	3	2	3	8
Carta 2018	2	1	3	6
Chiesa Estomba 2015	3	2	3	8
Chun 2010^a^	4	1	2	7
Karatzanis 2010^b^	3	2	3	8
Mendenhall 1996^c^	3	0	1	4
Oridate 2009^c,d^	4	0	1	5
Orus 2000^d^	4	1	1	6
Spriano 1997	4	2	3	9

^a^length of follow-up not reported, ^b^surgery only cohort, ^c^short follow up period, ^d^not all patients accounted for, ^e^radiation only cohort

### Aspiration

**Fig. 2:**
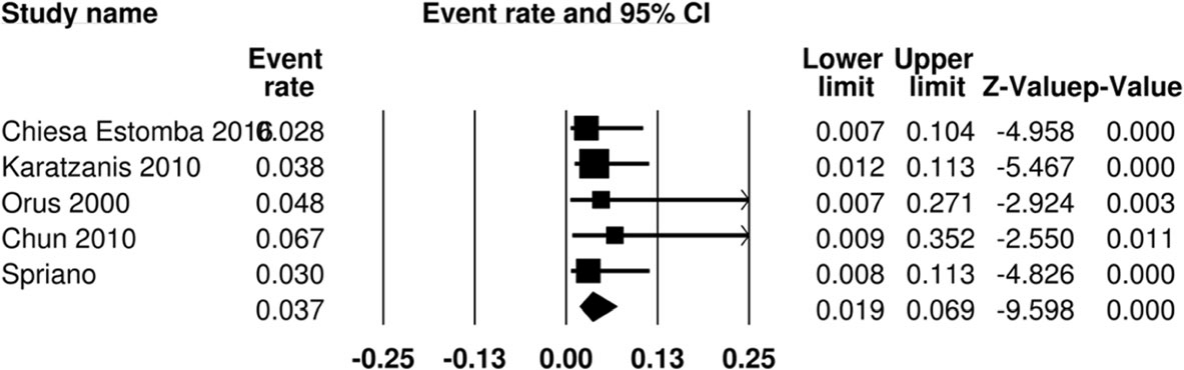
Pooled event rate of aspiration for early stage SSCC treated with organ preservation surgery

**Fig. 3:**
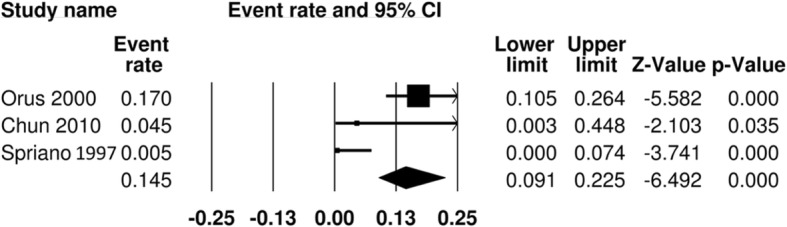
Pooled event rate of aspiration for early stage SSCC treated with radiation

**Fig. 4:**
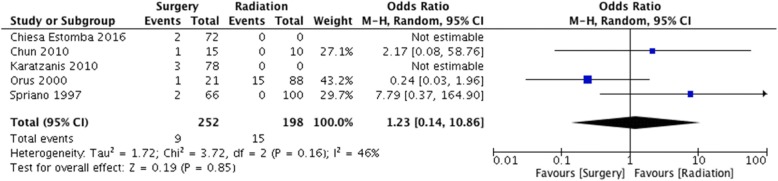
Forest Plot of comparison between organ preservation surgery and radiation with respect to aspiration events

Aspiration was deemed a primary functional outcome measure of assessment. It was reported in 5 studies [11, 14, 19–21]. In one paper, this was evaluated under direct visualization with a functional endoscopic examination of swallowing, where the authors documented liquid penetration. The remainder of the papers reported patients with clinically evident late aspiration, and sequelae such as aspiration pneumonia. In the surgical arm, 9 out of 252 patients were reported to experience aspiration, for a pooled aspiration rate of 3.7% (95% CI 1.9–6.9%) (Fig. 2)**.** In the RT arm, aspiration is reported in 15 out of 198 patients, for a pooled aspiration rate of 14.5% (95% CI 9.1–22.5%) (Fig. 3)**.** The odds ratio is 1.23 (95% CI 0.14–10.86) (Fig. 4).

### Intractable aspiration management

**Fig. 5:**
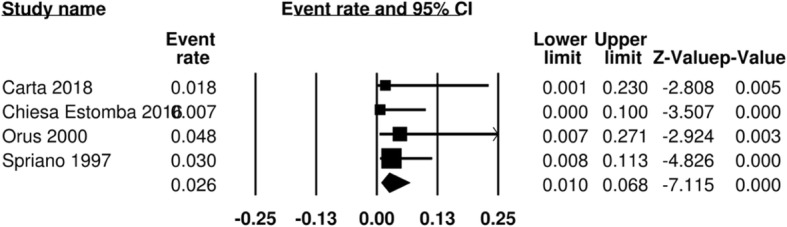
Pooled event rate of management of intractable aspiration for early stage SSCC treated with organ preservation surgery

**Fig. 6:**
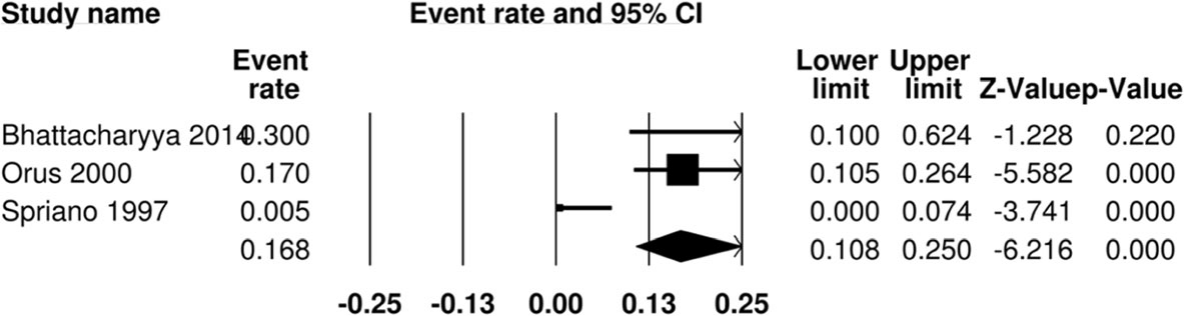
Pooled event rate of definitive aspiration management for early stage SSCC treated with radiation

**Fig. 7:**
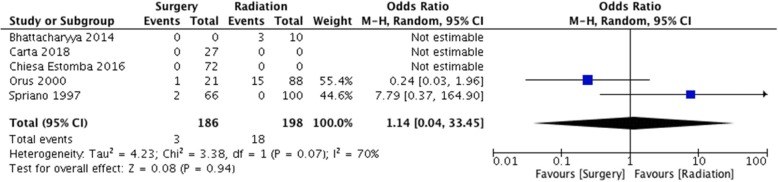
Forest Plot of comparison between organ preservation surgery and radiation with respect to management of intractable aspiration with permanent tracheostomy or functional laryngectomy

In many of the studies, rate of functional laryngectomy and permanent tracheostomy dependence was reported. These statistics included some, but not all, of the patients who experienced late complications associated with aspiration events. This was reported in 5 studies, accounting for 186 surgical patients and 198 radiation patients. The pooled event rate for intractable aspiration management in the surgical arm is 2.6% (95% CI 1.0–6.8%) (Fig. 5). The pooled event rate for the RT arm is 16.8% (95% CI 10.8–25.0%) (Fig. 6). The odds ratio comparing the two groups is 1.14 (95% CI 0.04–33.45) (Fig. 7).

### Permanent gastrostomy tube and swallowing dysfunction

**Fig. 8:**
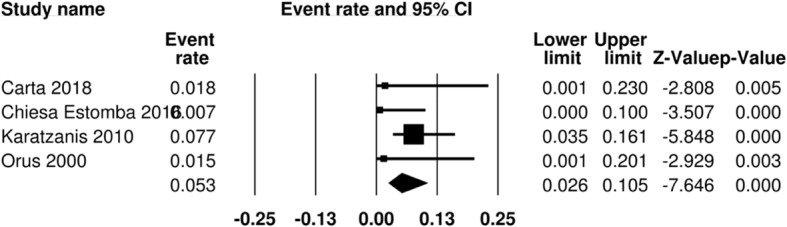
Pooled event rate of permanent gastrostomy tube for early stage SSCC treated with organ preservation surgery

The rate of permanent gastrostomy tube was reported in four studies for the OPS group. This accounted for 198 patients. This outcome measure was not reported for the radiation group in any of the studies. The pooled event ratio for permanent gastrostomy tube dependence is 5.3% (95% CI 2.6–10.5%) (Fig. 8).

Additionally, Chun et al. compared OPS to RT with respect to functional outcomes of swallowing and speech. To evaluate swallowing, patients underwent a functional endoscopic examination of swallowing (FEES) using videoesophagofluoroscopy. The researchers looked specifically for evidence of aspiration. Liquid aspiration was identified 6% of patients in the OPS group (*n* = 1). There was no identified liquid aspiration in the RT cohort [11].

### Quality of life and voice outcomes

Quality of life data was reported in one study. Another study reported objective voice outcome measures. Oridate et al. compared VRQOL, VHI-10, and GRBAS scores for T2 N0 SSCC against T1a, T1b, and T2 N0 glottic carcinomas and found no significant differences in functional outcomes [22].

Chun et al. used stroboscopy and acoustic waveform analysis to objectively evaluate voice outcomes. Abnormalities were in mucosal waveforms were identified 13% of the OPS cohort (*n* = 2) and 20% of the RT group (*n* = 2). These findings were not statistically significant [11].

## Discussion

To our knowledge, this is the first systematic review evaluating the functional outcomes of surgery versus radiotherapy for early-stage SSCC. All studies that met inclusion criteria were retrospective in design and there were 3 head-to-head comparisons of surgery versus radiation. Aspiration events, management of intractable aspiration, and permanent gastrostomy tubes are some of the major indications of laryngeal function. There was insufficient data to thoroughly meta-analyze the two modalities with respect to these outcome measures. Furthermore, objective voice outcomes were available in just two studies. Quality of life measures were only found in one included paper.

Aspiration events were reported, in many cases, with little additional information. In some studies, these were clinically evident respiratory events, such as aspiration pneumonias that occurred late in the post-operative course, others reported only the number patients that experienced aspiration. Aspiration events are reported in 50% of studies (*n* = 5), 2 of which were head-to-head comparisons.

For intractable aspiration intervention, there was data from both the surgical and radiation cohorts from multiple studies. This was reported as permanent tracheostomy dependence or conversion to a functional total laryngectomy. We calculated pooled event rates as well as pooled odds ratio. From the current data available, there is no strong evidence to suggest there is a difference between the two modalities with respect to this outcome measure.

With respect to permanent gastrostomy tube rate, there was no available data for the radiation cohort. Four papers reported this outcome measure for the surgical group (*n* = 198 patients). As a result, we were unable to make comparisons across the two treatment modalities.

Subjective and objective measures of voice outcome were sparsely and inconsistently reported. Quality of life measures were found in just one study, which actually compared T2 N0 supraglottic cancers to early glottic cancers.

### Surgery and radiotherapy for early stage SSCC

Several factors are important in considering treatment options for patients with early laryngeal cancer. Survival outcomes are obviously at the forefront. While there is no RCT data comparing survival outcomes of patients with early SSCC, a 2016 meta-analysis by Patel et al. studying early stage SSCC suggests that patients who undergo primary surgery have better survival than those who underwent primary radiotherapy [8]. Laryngectomy rate is another important consideration, as most patients with early stage laryngeal cancer are candidates for organ preservation treatment. Finally, in the presence of equivalent survival for two treatment modalities, functional outcomes are important to assess in comparing treatment options.

Over the last 40 years the treatment of early stage laryngeal cancer has evolved [23, 24]. Surgery, in the form of open partial laryngectomy, was initially popular however many patients had poor functional outcomes including aspiration and impaired base of tongue and laryngeal movements leading to swallowing dysfunction [25]. Radiation then took over as the primary treatment modality for early stage laryngeal cancer, with the goal of mitigating many of the functional problems associated with open partial laryngectomy. However, radiation is not without its own side effects. In addition, minimally invasive surgical techniques including TORS and TLM are increasingly being used for early stage SSCC [23, 24], raising the question of whether the functional outcomes with TORS and TLM may be better than radiation. Unfortunately, there are very few studies reporting the functional outcomes of TORS and TLM for early stage supraglottic cancer.

There are several advantages of RT. It preserves the laryngeal structures, it is generally well tolerated by patients, and increases surgical exposure to achieve excellent oncologic outcomes [26–29]. Radiation provides an effective treatment modality for patients not considered candidates for OPS due to their comorbid medical conditions. One of the disadvantages in treating early stage SSCC with RT, is that these patients are at high risk for developing a second primary and local regional recurrence [5, 6, 30]. If radiation is used as the primary treatment modality, most patients can only be salvaged with surgery, and, in the case of recurrent or new laryngeal cancer, the treatment is almost always total laryngectomy.

Surgical approaches include open surgery or transoral surgical approaches, including laser (TLM) and robotic (TORS). Organ-preserving surgery, both open and endoscopic approaches, offer several advantages over RT. As mentioned, patients with SSCC have reasonable 5-year overall survival rates, albeit with an increased risk of developing second primary aerodigestive tract malignancy [5, 6, 30]. Surgery can therefore be utilized as the first line, and, in the setting of recurrence, radiation can be used as salvage therapy. An additional advantage of surgery is the cost benefit of surgical intervention over radiotherapy [31, 32]. Furthermore, surgical management with a neck dissection provides the opportunity to identify occult metastasis; an important consideration given that up to 30% patients with SSCC may have occult nodal metastasis [32]. Identification of occult metastasis allows for accurate staging of patients, and subsequently treatment with multimodality therapies.

Dombree et al. analyzed the cost of open supraglottic laryngectomy, TLM, and TORS in a Belgian model. Their study suggests the cost of open supraglottic laryngectomy similar to that of TLM in upfront surgical costs [31]. TORS tends to be more expensive primarily due to purchase and maintenance costs [31]. This study did not account for in-hospital costs such as length of admission, complications or readmission rates. With regard to glottic cancers, a cost analysis was carried out for a Canadian model comparing TLM to radiotherapy. This showed TLM to be a more cost-effective treatment option [33].

There are also disadvantages of surgery, including risk of general anesthetic, particularly in patients with comorbidities, bleeding, and infection. Pharyngocutaneous fistula, dysphagia and permanent tracheostomy dependence are specific risks of supraglottic laryngectomies. A criticism of OPS are the associated poor functional outcomes [25]. However, newer surgical techniques including TLM and TORS have gained popularity recently, and hold several advantages over open surgery and RT. In one study, TLM was compared to open surgery, resulting in reduced incidence of permanent gastrostomies and tracheostomies [20]. Since the introduction of TLM by Strong and Jako, there have been several reports investigating the role of TLM for supraglottic laryngectomy [34–48]. With respect to TORS, the majority of the studies report on all stages of supraglottic SCC [20, 43, 48–59]. With that in mind, long-term tracheostomy and gastric feeding tube rates range from 0 to 20% in patients treated with TORS [49, 52, 55]. None of the studies included in this systematic review examined the functional outcomes of TORS.

### Strengths

There are several strengths to this review. To our knowledge, this is the first comprehensive review of all available literature comparing functional outcomes between surgery versus radiation for patients with early stage SSCC. It was designed, conducted and reported in accordance with published guidelines (PRISMA) and the study protocol, as well as search strategy, was outlined a priori. A comprehensive search strategy was undertaken and led to the review of 5218 unique citations of which ten studies met our inclusion criteria. This resulted in the analysis of a large number of patients with early stage SSCC (*n* = 640).

### Limitations

As with all systematic reviews, the strength of the conclusions that can be drawn from this study depend on the quality of the primary studies. The included studies were evaluated with the Newcastle-Ottawa Scale for Assessing Cohort Studies, most of which were of moderate in overall quality. Next, although we only included studies published from 1990 forward, some of the studies in our review included patients treated well before that time period. This broad recruitment period includes many patients where treatment algorithms may not reflect today’s standards. Given the improvements in medical imaging, some patients may have had regional nodal disease which was not evident on the available scans, therefore reflecting more advanced disease. Furthermore, many of the current treatment options, such intensity modulated radiotherapy (IMRT), TLM, and TORS were not in clinical practice prior to 1990. The types of radiation and protocols used were not clearly outlined in the studies included. As well, given the broad recruitment periods for some of these studies, different radiotherapy protocols would have been offered to the patients according to the available therapies at that time.

All 10 studies that met the inclusion criteria were retrospective study designs and there were no randomized controlled trials. Retrospective studies have inherent biases including selection biases. Patient’s with medical comorbidities may not have been deemed appropriate surgical candidates and only offered radiotherapy, which may not be reflected in the results.

Significant heterogeneity was noted between the outcome measures of the included studies. In the surgical group, not all patients may have received the same type or extent of surgery, including elective neck dissections, TLM, and TORS operations. We only considered English language studies for our systematic review, which limited the number of titles screened and studies included, however, the effect of this would likely be small.

The heterogeneity of the functional outcomes reported limited our ability to meta-analyze the data. Additionally, many of the outcomes were sparsely reported or reported for only one of the arms of study, either OPS or RT. The overall paucity of data limits our ability to draw conclusions.

Given the lack of high level evidence guiding the optimal management of early stage supraglottic cancer and potential biases of retrospective studies, a head to head comparison between newer modalities such as TLM and TORS with RT is critical in determining the therapeutic algorithm that can yield better functional outcomes in early stage SSCC patient. Many studies were ruled out as part of our protocol due to the stratification of results with respect to laryngeal subsite and staging criteria (including advanced stage disease). The method in which functional results are reported, is often less rigorous than the reporting of survival and locoregional control. The use of objective measures and validated tools was limited and not consistent across studies.

Traditionally, studies comparing surgery and radiation have been challenging to accrue patients to. Ongoing efforts comparing OPS to RT for oropharyngeal cancer are underway and actively accruing [60, 61], demonstrating that a head-to-head comparison of surgery and radiation is a possibility for patients with early stage supraglottic cancer. High level of evidence is important in the development of treatment guidelines for patients with early stage disease that have a surprisingly poor prognosis, compared to other early stage head and neck cancers. Traditionally, OPS has been seen as an option with poor functional outcomes. We did not find evidence to support one modality being better than another with respect to functional outcomes.

## Conclusions

There is an overall paucity of literature available regarding functional outcomes in the treatment of early stage SSCC patients, the majority of which is moderate in overall quality and retrospective in nature. The majority of the data does not reflect the treatment algorithms of today, with the introduction of focused radiation therapies and endoscopic surgical approaches. Changes to treatment algorithms have not been reflected in head-to-head studies examining the functional outcomes. The outcome measures reported have significant heterogeneity in the current literature, which limits the ability to draw definitive conclusions.

Moving forward, we propose that aspiration, intractable aspiration intervention, permanent gastrostomy tube requirements, and objective quality of life scales as objective measures that should be included in future research on this topic. Future studies and research should include well designed prospective trials with rigorous reporting of outcome measures.

## Abbreviations

CI: Confidence Intervals
FEES: Functional endoscopic examination of swallowing
NCCN: National comprehensive cancer network
ND: Neck dissection
NR: Not reported
OPS: Organ preservation surgery
OR: Odds Ratios
PRISMA: Preferred Reporting Items for Systematic Reviews and Meta-Analyses
RT: Radiation Therapy
SSCC: Supraglottic squamous cell carcinoma
TLM: Transoral endoscopic laser microsurgery
TORS: Transoral robotic surgery
